# Procedural complications of inferior vena cava filter retrieval, an illustrated review

**DOI:** 10.1186/s42155-020-00113-6

**Published:** 2020-04-27

**Authors:** Keith B. Quencer, Tyler A. Smith, Amy Deipolyi, Hamid Mojibian, Raj Ayyagari, Igor Latich, Rahmat Ali

**Affiliations:** 1 Division of Interventional Radiology, Department of Radiology, University of Utah, 50 N Medical Drive, Salt Lake City, UT 84132 USA; 2 Division of Interventional Radiology, Department of Radiology, Memorial Sloan Kettering, New York, USA; 3 Division of Interventional Radiology, Department of Radiology, Yale University, New Haven, USA

**Keywords:** IVC filter removal, Bronchial forceps, Sling technique, IVC filter leg penetration, Tilted IVC filter

## Abstract

Annually, approximately 65,000 inferior vena cava (IVC) filters are placed in the United States (Ahmed et al., J Am Coll Radiol 15:1553–1557, 2018). Approximately 35% of filters are eventually retrieved (Angel et al., J Vasc Interv Radiol 22: 1522–1530 e1523, 2011). Complications during filter retrieval depend heavily on technique and filter position. In this paper, we review risk factors and incidence of complications during IVC filter removal. We also discuss ways these complications could be avoided and the appropriate management if they occur.

## Background

Inferior vena cava filters are employed in a variety of clinical scenarios (Table 1) (DeYoung and Minocha 2016). Complications of in situ filters include cardiac migration, leg fracture with or without embolization, caval thrombosis and symptomatic caval penetration by filter legs (Grewal et al. 2016). Because of the increased recognition of in situ filter complications and advances in retrieval techniques, filter retrieval rates have increased over the years (Angel et al. 2011; Ahmed et al. 2018) To avoid complications of in situ filters, removal is indicated when the filter is no longer needed (Table 2). Maintenance of patients on therapeutic anticoagulation at the time of retrieval is recommended (Kaufman et al. 2006) and is not associated with increased risk of retrieval complications (Schmelzer et al. 2008).

**Table 1:** Indications for IVC filter placement ^a^Filter used as an adjunct to anticoagulation

Acute VTE with contraindication to anticoagulation	
Failure of anticoagulation in the setting of VTE	
Hemodynamic instability in patients with acute VTE^a^	
Massive PE being treated with thrombolysis or thrombectomy^a^	
Mobile iliocaval DVT^a^	
Prophylaxis after major trauma or prior to obesity surgery

**Table 2:** Indications for Filter Removal (Kaufman et al. 2006)

Risk of PE is low (patient is anticoagulated or clinical status has changed)	
Anticipated patient survival is > 6 months	
Filter can be removed safely	
Future return to need for filter is not anticipated (e.g. *major* upcoming surgery not planned)	

The reported procedural complication rates of filter retrieval range from 0% (Tashbayev et al. 2016) to 20% (Brahmandam et al. 2019). Complication rates depend on multiple factors including whether advanced techniques were used and filter position (Table 3). In this paper we examine risk factors for complications from filter retrieval, discuss approaches to avoid such complications and provide possible treatment approaches for when complications do occur.

**Table 3:** Reported major procedural complication rates during IVC filter removal

Removal technique	Major complication rates	Complication Description
Standard loop snare	0% (Asch 2002; Tashbayev et al. 2016; Zakhary et al. 2008) (Ahmed et al. 2020) 0.4% (Brahmandam et al. 2019) 5% (Al-Hakim et al. 2014)	Access complications (pneumothorax, jugular vein thrombosis) (Terhaar et al. 2004)
Bronchial forceps	0.8% (Stavropoulos et al. 2015) 6.7% (Tavri et al. 2019) 8.3% (Lian et al. 2019) 11.8% (Al-Hakim et al. 2014)	Leg fracture with embolization (Lee et al. 2018; Poliwoda et al. 2019). Contrast extravasation/RP hemorrhage (Lian et al. 2019). Renal artery to IVC fistula (DeSai et al. 2019; Ferral 2019)
Laser-assisted removal	0% (von Stempel et al. 2019) 1.6% (Kuo et al. 2017) 3% (Kuo et al. 2013)	Caval thrombosis, caval injury with hemorrhage needing stent graft placement
Sling technique	0% (Rubenstein et al. 2007) 1.9% (Al-Hakim et al. 2014) **20%** (Brahmandam et al. 2019)	IVC dissection, contrast extravasation, strut fracture

## Main text

### Factors predisposing to complications

#### Procedure technique

Advanced retrieval techniques are defined as anything beyond simple snare of the filter hook with subsequent over-sheathing. Advanced techniques are used in cases when snaring the hook is not possible (e.g. when the filter hook is embedded in the caval wall) or when the filter’s legs have become embedded. Advanced techniques include endobronchial forceps assisted retrieval (Stavropoulos et al. 2015), the sling technique (Rubenstein et al. 2007)**,** endovascular laser sheath removal (Kuo et al. 2017) and centering techniques using balloons or flossing techniques (Lynch 2009). These advanced techniques may be combined. Compared to simple removal, the procedural complication rates are significantly higher when advanced techniques are used; studies have shown a 4-fold increase in overall complications (5% to 20%)(Brahmandam et al. 2019) and a 13x increase in major complications (0.4% to 5.3%) (Al-Hakim et al. 2014). Therefore, when employing advanced techniques, it is recommended to have a semi -compliant tamponade balloon, such as a 32 mm the CODA balloon (Cook Medical Bloomington, IN) as well as appropirately sized bare metal stents and stent grafts immediately available. Even when complication free, advanced techniques are associated with 5.4x-more fluoroscopy time (23.1 vs 4.3 min) and a 3.6x greater radiation exposure (557.2 vs 156.9 mGy) (Ahmed et al. 2020).

##### Endobronchial forceps

**Fig. 1:**
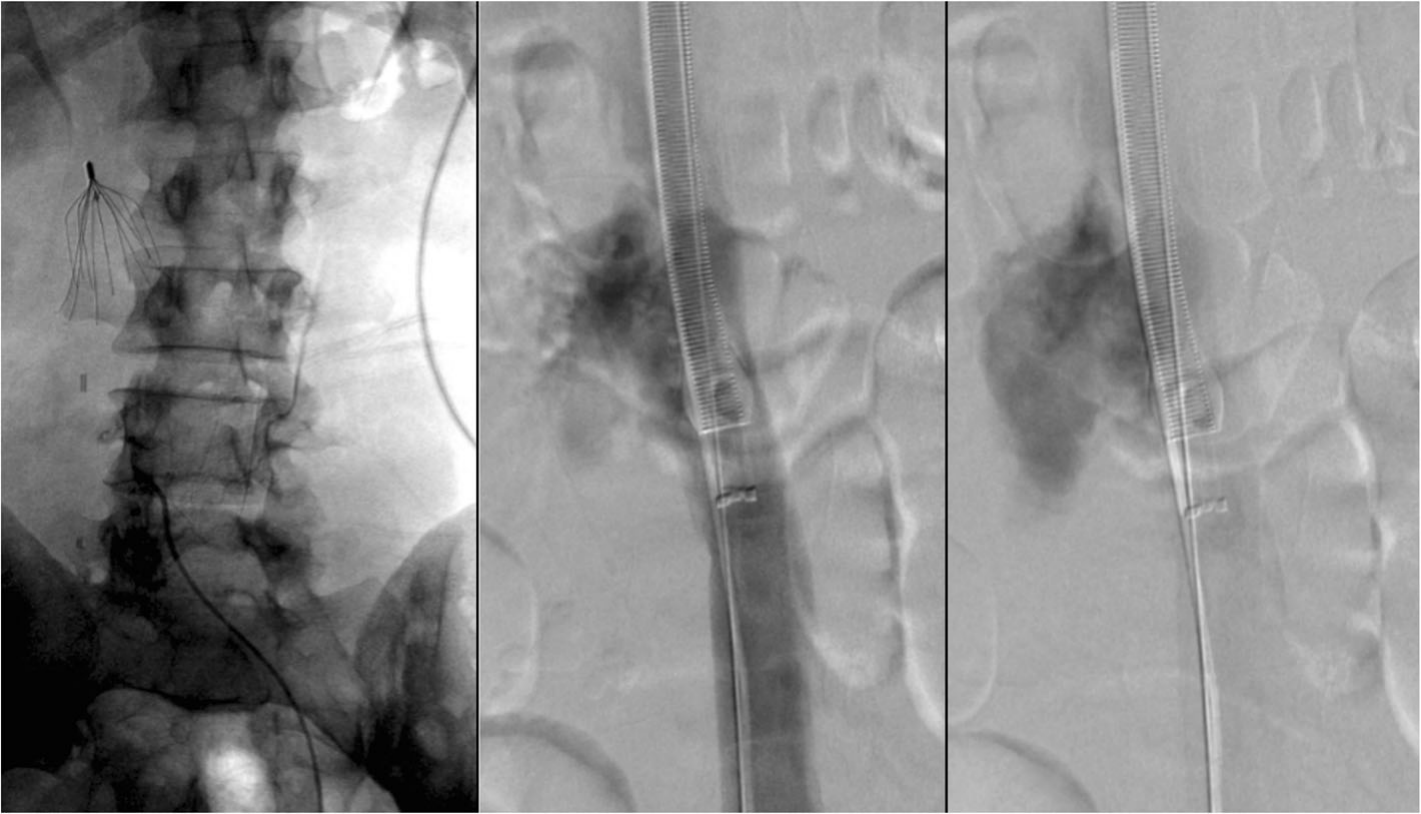
Patient with a Bard Recovery Filter (Bard Peripheral Vascular, Inc., Tempe, AZ) placed at an outside hospital 11 months prior presented for filter removal. Bronchial forceps were used because significant filter angulation prevented use of the cone recovery system. Follow-up cavagram after showed significant contrast extravasation. Laboratory evaluation 1 h post procdure procedure revealed a 7 point HCT drop and the patient was admitted overnight for close observation

**Fig. 2:**
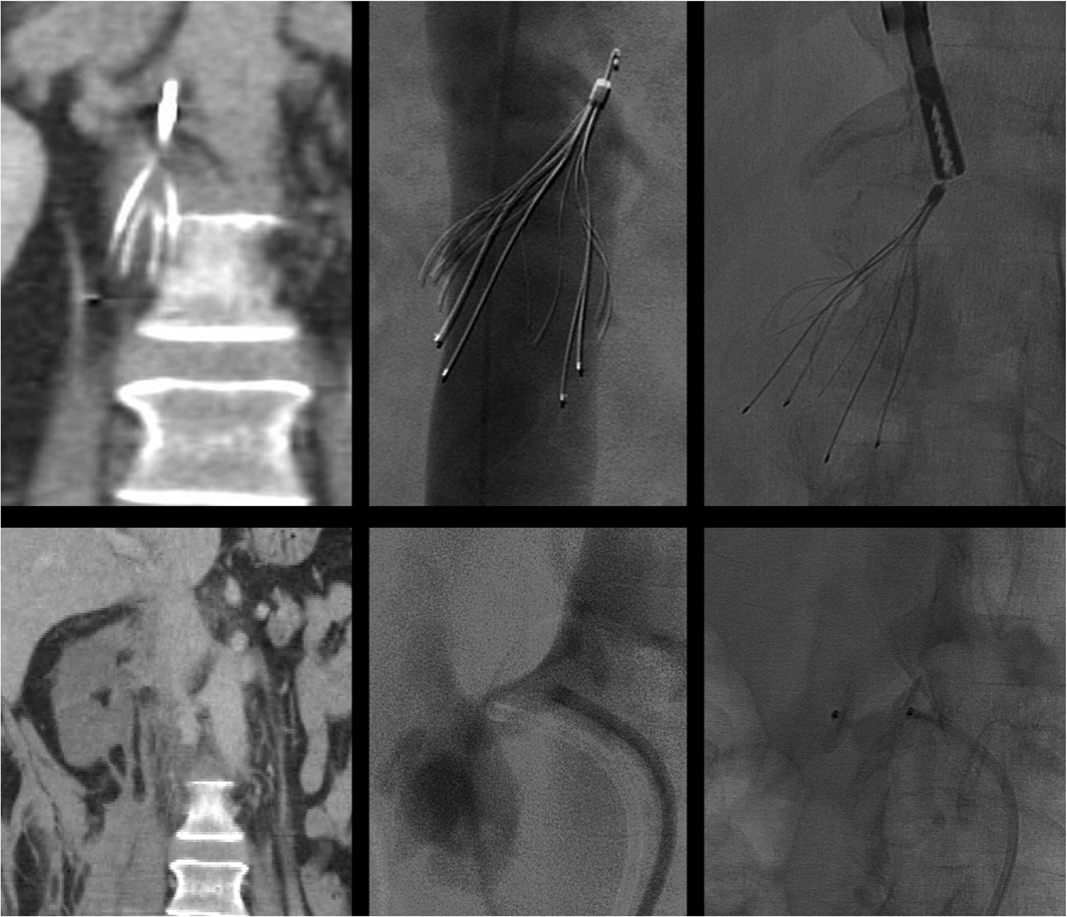
This patient had undergone placement of a Cook Celect filter (Cook, Bloomington, IN) 24 weeks prior to presentation for filter retrieval. Pre-procedure CT showed the right hook of the IVC filter to be protruding into the right renal artery. Cavagram showed an angulated filter with an embedded hook and therefore bronchial forceps were used. After technically sucessful filter retrieval, the patient developed hypotension and right sided flank pain. CT showed a right sided retroperitoneal hemorrhage and an infarcted right kidney. Angiogram was done demonstrating a fistula between the right renal artery and IVC and the renal artery was embolized with a 12 mm Amplatzer Vascular plug. In this case, with pre-procedure CT evidence of the hook penetrating into the renal artery, obtaining arterial access could have been done to allow imaging and potential treatment of any renal arterial disruption during retrieval

**Fig. 3:**
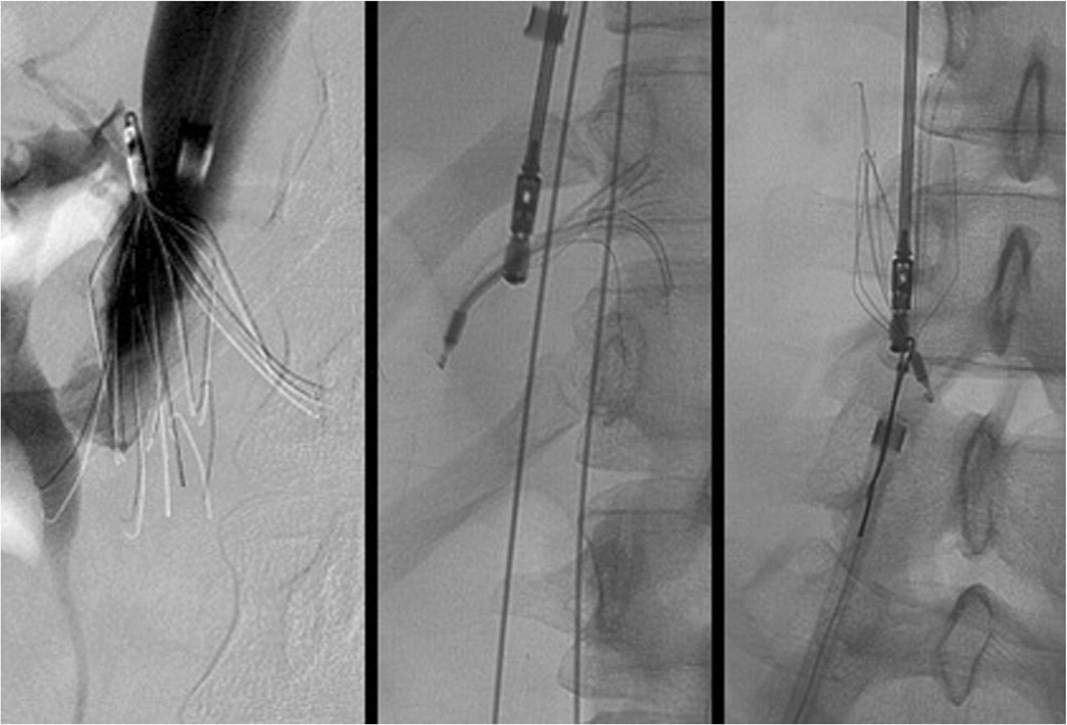
Initial cavagram showed significant rightward filter tilt with penetration of legs and hook of this Bard Eclipse filter (Bard Peripheral Vascular, Inc., Tempe, AZ). Bronchial forceps were used to grasp the neck of the filter. This resulted in unintentional tilt of the filter and distortion of the filter. The filter was inverted and the neck was snared and pulled into the external iliac vein. The filter could not be removed endovascularly and the patient was taken urgently to the operating room for open femoral vein cut down for extraction

Endobronchial forceps are employed in cases where the hook of the filter is not accessible to snaring, most often because of significant tilt, embedded hook, or a fibrin cap covering the filter hook. In these cases, rigid bronchoscopy forceps dissect the hook of the filter from the caval wall. In the largest series to date (which included 114 patients), the only major complication reported was a symptomatic IVC pseudoaneurysm requiring balloon tamponade and a 2 night hospital admission. The same series also reported 3 minor complications, including two filter leg fractures with embolization to the pulmonary artery, which were sucessfully snared. The other minor complication was an asymptomatic IVC pseudoaneurysm (Stavropoulos et al. 2015). Trauma to the IVC will occur if the operator inadvertently grasps the caval wall (Daye and Walker 2017) with one series showing imaging evidence of contrast extravasation in 8.3% of bronchial forceps filter removals (Lian et al. 2019) (Fig. 1). Traumatic arterio-venous fistula between the renal artery and IVC after foceps filter removal have been reported (DeSai et al. 2019; Ferral 2019) (Fig. 2). Additionally, multiple case reports of leg fractures with embolization have been reported (Knavel et al. 2016; Lee et al. 2018; Poliwoda et al. 2019). Other complications from utilizing the bronchial forceps may arise (Fig. 3).

##### Sling technique

**Fig. 4:**
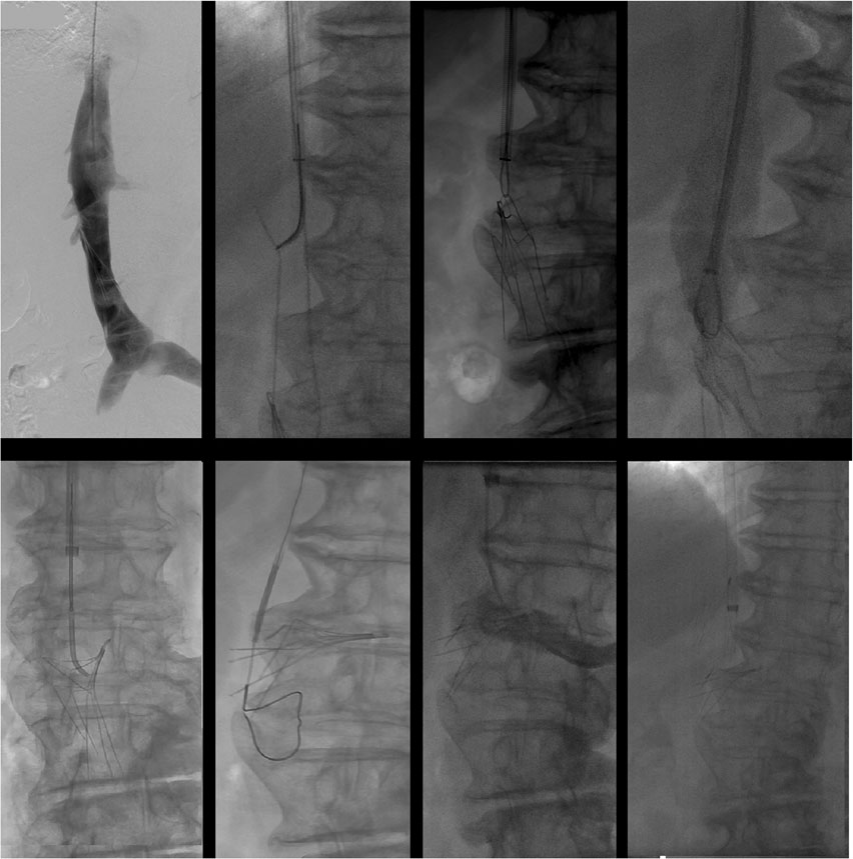
This patient had an Argon Option Elite filter (Argon Medical Devices, Plano, TX) placed 3 months prior to attempted retrieval. Significant rightward apical tilt was seen on initial venogram retrieval precluding snaring of the hook. The sling technique was then employed. The filter was then pulled cranially resulting in significant leftward tilt and eventually entered the left renal vein. This caused acute left renal vein thrombosis. A suprarenal filter was placed. 3 main lessons can be gleaned from this case: 1-filters should not be “pulled” but rather should be over sheathed, 2-when using the sling technique, at least two legs should be engaged, 3-consideration should be given to using the modification of the sling technique where-by, the sling is used only to realign the filter allowing the hook to become accessible and subsequently snared in a standard fashion

**Fig. 5:**
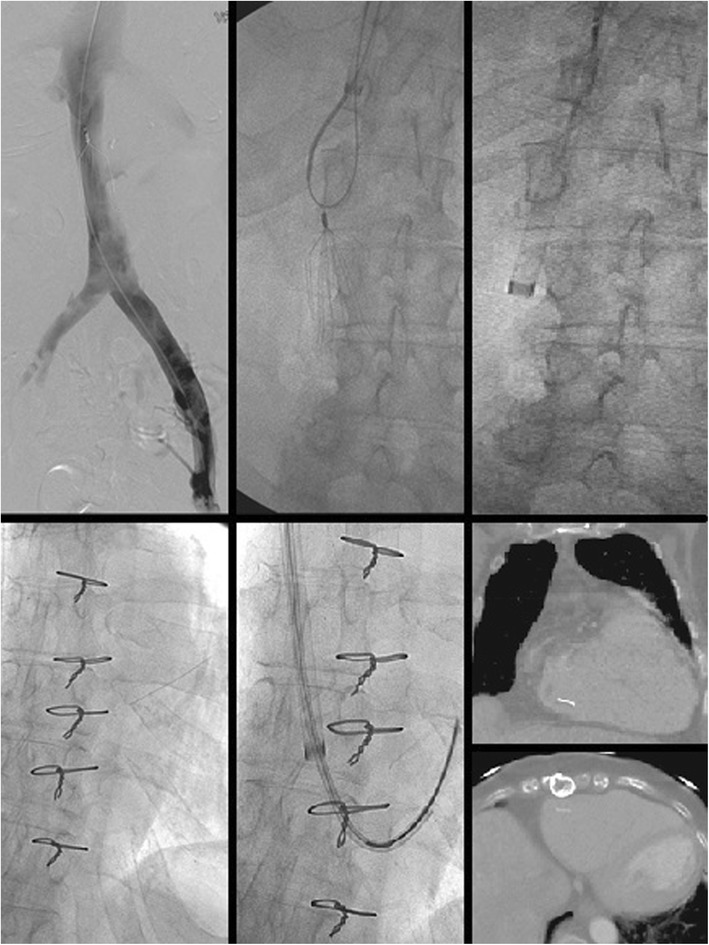
Initial cavagram shows an intact Bard Eclipse filter (Bard Peripheral Vascular, Inc., Tempe, AZ) IVC filter. Simple loop snare was unsucessful because of posterior tilt and embedded hook. The Hangman’s technique, a modification of the sling technique where a glidewire loop is created using a reversed curved catheter is created between the caval wall and filter hook to separate the tip of the filter from the wall of the filter, was used. After sucessful filter removal, ex-vivo examination showed two missing filter legs. Subsequent images showed one piece to be in the pulmonary outflow tract and one in the right atrium. The larger fragment was sucessfully retrieved from the pulmonary outflow tract but the piece in the right atrium could not be removed. Subsequent echocardiogram showed new severe tricuspid regurgitation which was managed medically. In situ leg fracture of certain types of filters is higher than others. The older generation Bard filters have a 25% rate of in situ leg fracture (Nicholson et al. 2010). While no published data exists on differential rates of fracture during retrieval by filter type, it is likely that filters prone to spontaneous in situ fracture are also more likely to fracture when subjected to mechanical stresses during retrieval

**Fig. 6:**
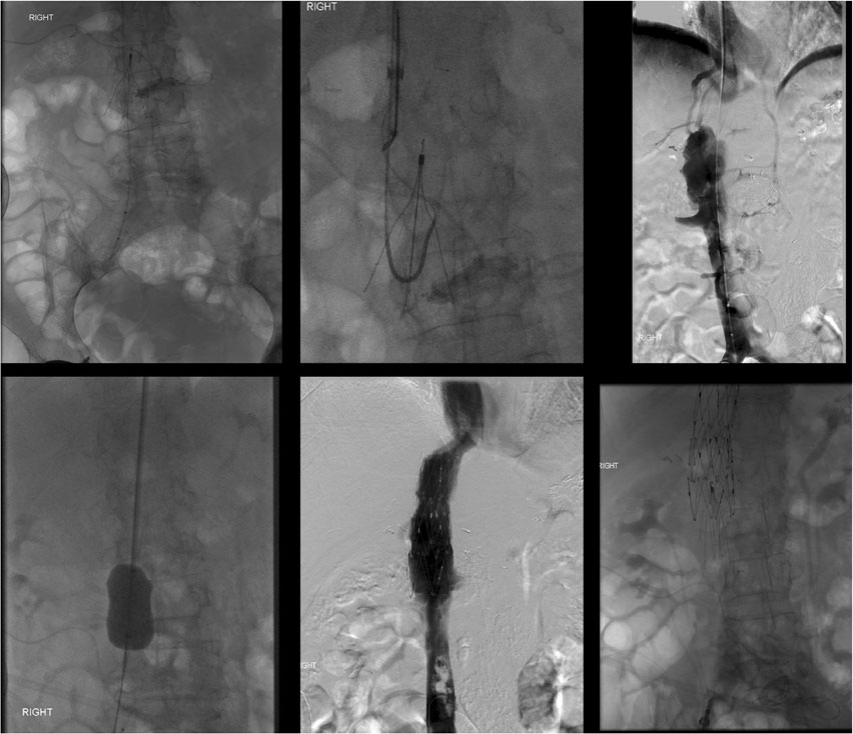
This patient had a Gunther-Tulip IVC filter (Cook, Bloomington, IN) placed 4 months prior to removal. The sling technique was employed given significant filter tilt. After removal, the patient developed abdominal pain and hypotension. Venogram showed extravasation of contrast and a flow limiting dissection. Balloon tamponade was performed but failed to resolve extravasation. Two overlapping 25 mm Cook-Z Stents (Cook, Bloomington, IN) were placed with improvement of flow and resolution of extravasation. In cases of venous extravasation, uncovered stents can often resolve extravasation (Funaki et al. 1997). Partial caval thrombosis secondary to stasis from balloon tamponade developed and a new IVC filter was placed above this clot

The sling technique (also known as the loop-snare technique) is also used in cases of embedded or angulated hooks. After a reverse-curved catheter placed below the filter, a glide wire is introduced through the legs of the filter, snared and externalized. After confirmation that at least 2 legs have been captured, back tension is applied to both ends of the wire while advancing the sheath over the filter. If the filter is pulled cranially, especially if only one leg has been captured, undesirable re-alignment of the filter can result (Fig. 4). Undesired re-orientation is also known to occur with the sling technique, especially with malleable nitinol filters (Kuyumcu and Walker 2016). A modification of this technique can be used to realign the filter into an upright position which is then followed by loop snaring of the hook. When this modification was employed in a study of 20 consecutive patients no complications occurred (Su et al. 2019), however other series have reported an almost 20% overall complication rate (Brahmandam et al. 2019). Filter leg fracture (Fig. 5) and IVC dissection with contrast extravasation (Fig. 6) have all been desribed as potential complications of this filter removal technique (Brahmandam et al. 2019).

##### Excimer laser

**Fig. 7:**
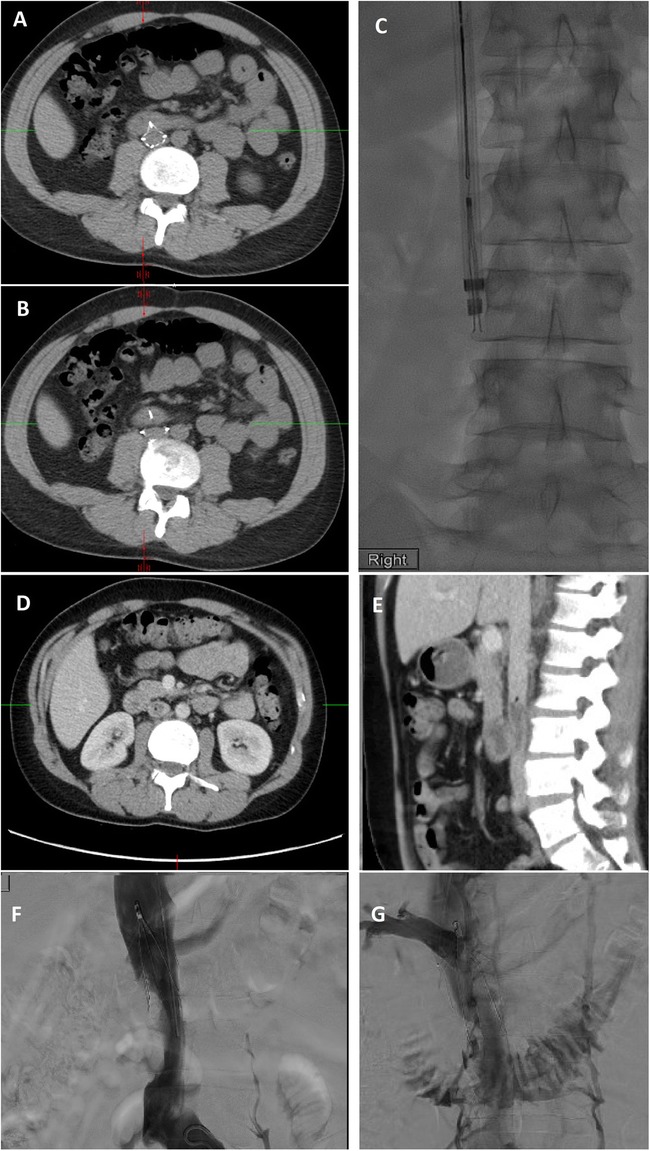
This patient had a Günther Tulip IVC filter (Cook, Bloomington, IN) for PE prophylaxis after a motor vehicle accident and was lost to follow-up until imaging was done for unrelated reasons 10.5 years later. Pre-retrieval CT (**a** + **b**) showed penetration of 2 legs of the filter into the doudenum. Given embedded legs, a a 14Fr Excimer laser sheath was employed (**c**). The day after retrieval, the patient developed fevers and 8 days later he underwent CT of the chest, abdomen and pelvis. Chest CT showed multiple cavitary nodules, pulmonary emboli (not shown) and new IVC clot with a small focus of gas at the cranial aspect of the clot (**d** + **e**). After filter placement cranial to this clot (**f**) and subsequent clot aspiration 20 French FlowTriever® Aspiration device (Inari Medical, Irvine, CA), cavagram showed a fistula to the doudenum (**g**). After the patient was placed on antibiotics and anticoagulation, bacteremia and IVC clot resolved. A case of a doudenal-caval fistula caused by an in-situ IVC filter has been reported in the literature (Vandy et al. 2011). Doudenal-caval fistula or septic VTE have not yet been described after filter removal, however

The Excimer laser was has been used for removal of filters whose legs have been incoperated into the IVC and cannot be removed by conventional means. A 12, 14 or 16Fr laser sheath is advanced through a 16 or 18Fr sheath and over the filter. Short periods of controlled photothermal energy is used to ablate adhesive tissue around embedded parts of the filter. In one study of 100 patients, 7% of patients undergoing filter removal using the Excimer laser had imaging evidence of caval injury; two of these necessitated balloon tamponade of the IVC followed by stent-graft placement. Other caval injuries included venous pseudoanurysms (4%) and contrast extravasation (3%) (Kuo et al. 2013). In a subsequent paper by the same group which, included 251 patients, there was a lower rate of major complications (1.6%) (Kuo et al. 2017) Here we show a not yet described cases of caval-enteric fistula with septic caval thrombophlebitis after laser sheath removal of a filter with leg penetration. Attribution of this complication is likely a combination of both the leg penetration into bowel as well as vessel injury from laser removal (Fig. 7).

#### Filter position

Certain patient and filter related factors can make filter retrieval more difficult and potentially more dangerous (Table 4). These include filters with significant tilt, an embedded hook, significant penetration and prolonged dwell time. Consideration of obtaining a pre-retrieval CT is recommended in patients who have had their filters in place for > 180 days to help with procedural planning and informed discussion regarding risks of filter removal (Dinglasan et al. 2013); as pre-procedure CT was found to be highly predictive of difficult retrieval. An embedded tip was the most predictive of difficult retrieval followed by > 15^°^ filter angulation and finally by grade 2 (struts outside the IVC lumen) and grade 3 (struts inserting into adjacent organ or retroperitoneal structure) leg penetration.

**Table 4:** Risk factors for difficult filter retrieval

Tilt (> 5^°^)	
Tip embedded in IVC wall	
Significant leg penetration	
Prolonged dwell time	

##### Filter angulation

Filter angulation is highly predictive of difficult removal and need for advanced retrieval techniques (Clements et al. 2019). Filters tilted between 5 and 15^°^ have and estimated 2.4x chance of a difficult retrieval. Filters tilted > 15^°^ have between 7.9 and 33 times greater risk of being difficult to retrieve (Clements et al. 2019; Dinglasan et al. 2013). Filters, such as the Bard Denali (Bard Peripheral Vascular, Inc., Tempe, AZ), with lower rates of tilt (Bos et al. 2016) have been associated with shorter procedures, reduced fluoroscopy times and less need for advanced techniques (Ramaswamy et al. 2018). The Argon Option Elite IVC filter, on the other hand, has been shown to require more advanced retrieval techniques, higher failure rates and longer fluoroscopy times, possibly related to greater filter tilt (Neill et al. 2017).

##### Embedded tip

**Fig. 8:**
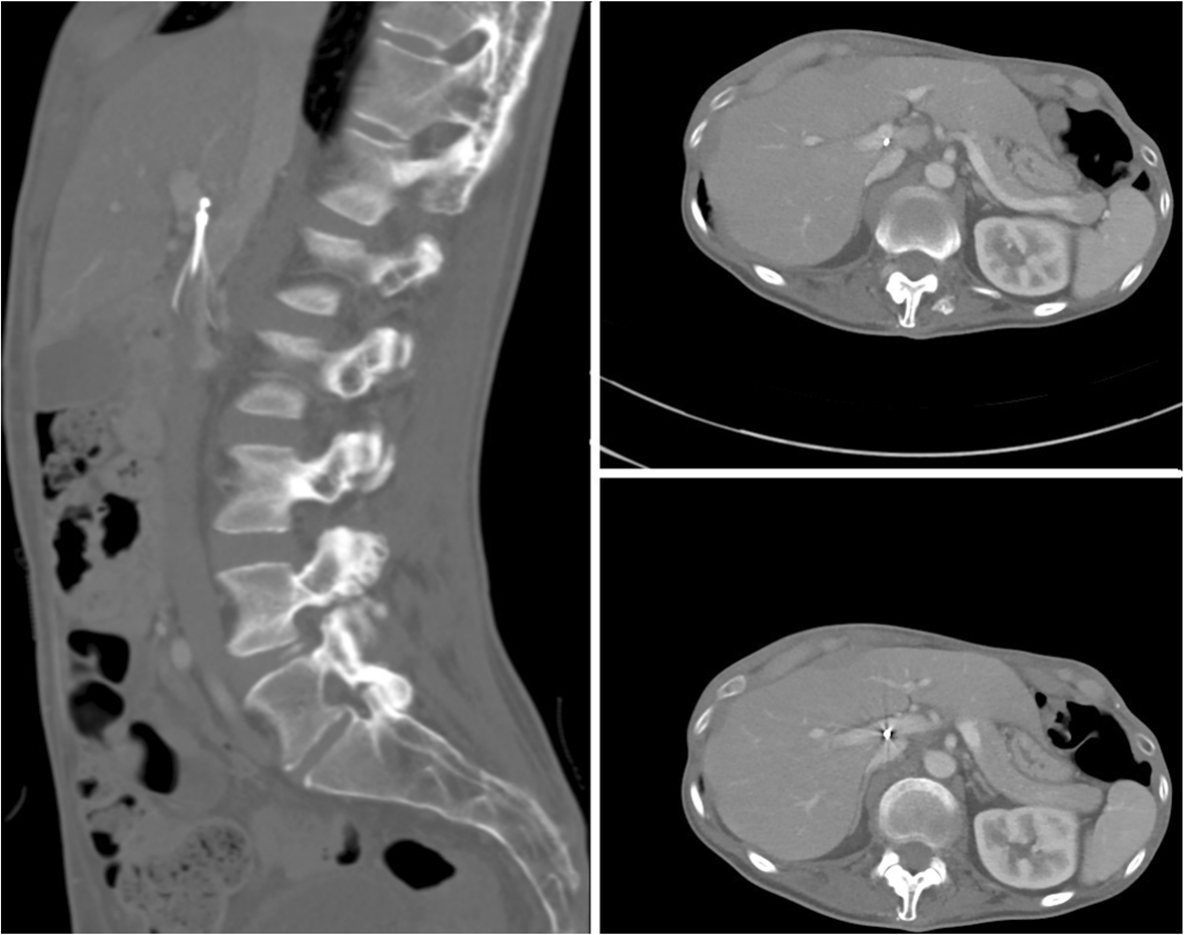
This patient had a suprarenal Günther Tulip IVC filter (Cook, Bloomington, IN) placed at OSH approximately 5 years prior to obtaining this CT. Images showed the filter hook outside the lumen of the IVC and within hepatic parenchemya and possibly into the portal vein. Given that the patient was asymptomatic and that filter removal could lead to signfiicant complications it was elected not to pursue retrieval

**Fig. 9:**
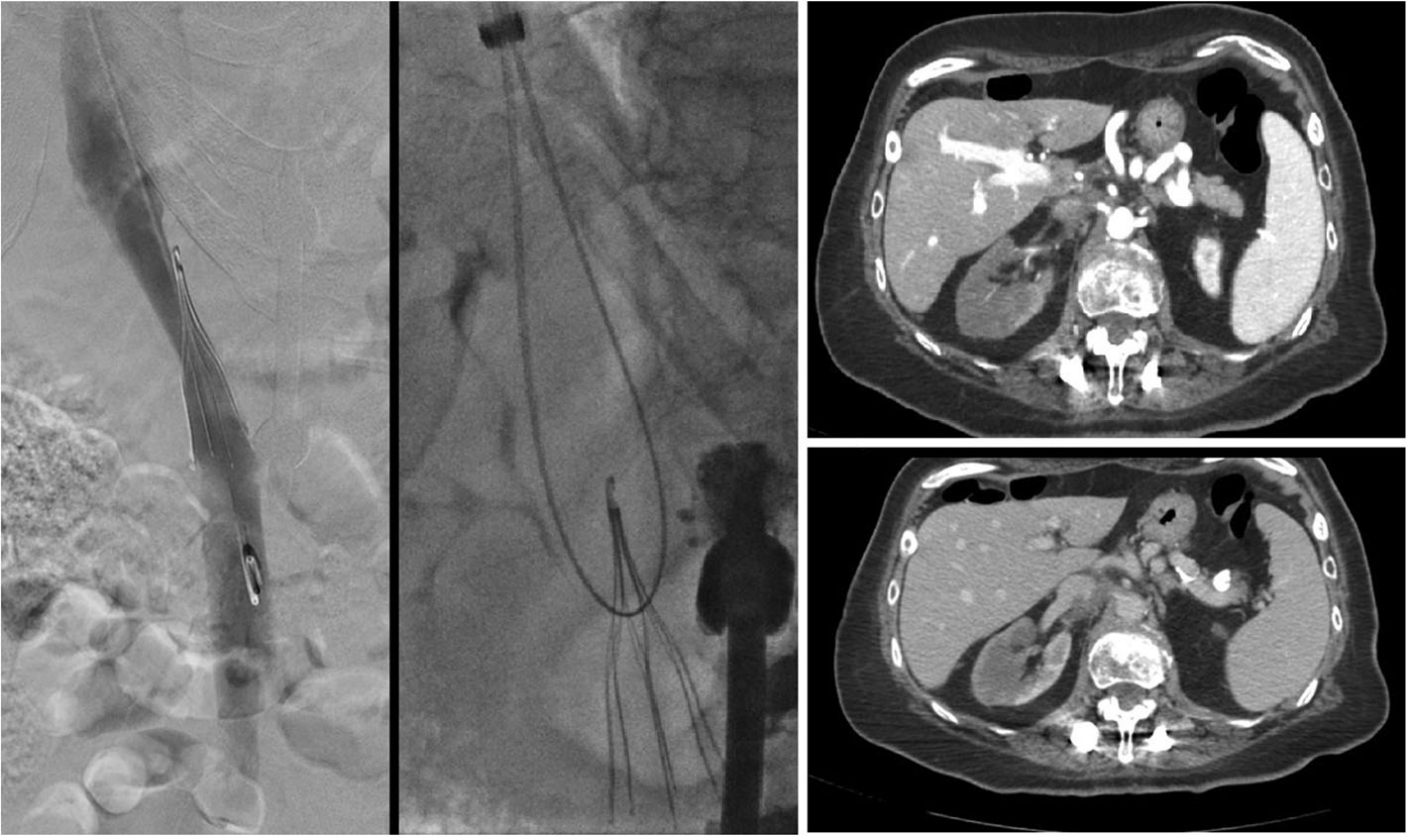
This patient had a pre-operative IVC filter (Argon Option Elite filter; Argon Medical Devices, Plano, TX) placed 3 months prior to attempted removal. Initial cavagram showed the tip to be embedded in the wall of the IVC at the junction of the left renal vein and IVC. Snaring was not possible, so the sling technique was employed. Three days later, the patient had persistent abdominal pain and underwent CT which showed a short segment renal artery occlusion, renal infarction and intramural hematoma of the IVC. Arterial injury after sling technique filter removal has not previously been described in the literature

Removal of a tip-embedded IVC filter is a high-risk procedure; the presence of an embedded tip is associated with an odds ratio of 129 for a complex retrieval (Dinglasan et al. 2013). A filter whose tip contacts the caval wall is prone to thrombus, neointimal hyperplasia, smooth muscle and dense fibrosis around the tip (Kuo et al. 2012; Singer and Wang 2011). Filter tips may also penetrate beyond the wall of the cava (Fig. 8). Removal of tip embedded filters is considered both difficult, because the hook is inaccessible to snaring and high risk because of need for “aggressive force” and use of advanced technique with potential damage to the caval wall during dissection of the embedded tip from the wall (Stavropoulos et al. 2008) (Fig. 9).

##### Leg penetration

**Fig. 10:**
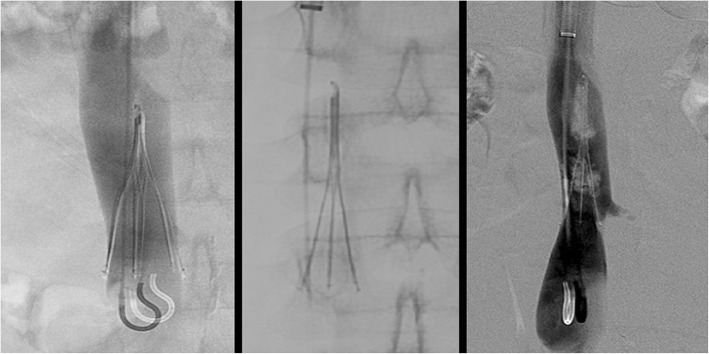
This patient had a prophylactic filter (Argon Option Elite IVC; Argon Medical Devices, Plano, TX) placed after an motor vehicle accident. The patient presented for retrieval 5 months later. Initial cavagram shows normal appearing and upright filter. The hook was easily snared but embedded legs precluded sucessful removal. Subsequent cavagram show non-occlusive thrombus and filter distortion. Partial caval thrombus was a frequent finding (31%) in patients who were undergoing filter retrieval with adherant legs (Kuo et al. 2009)

**Fig. 11:**
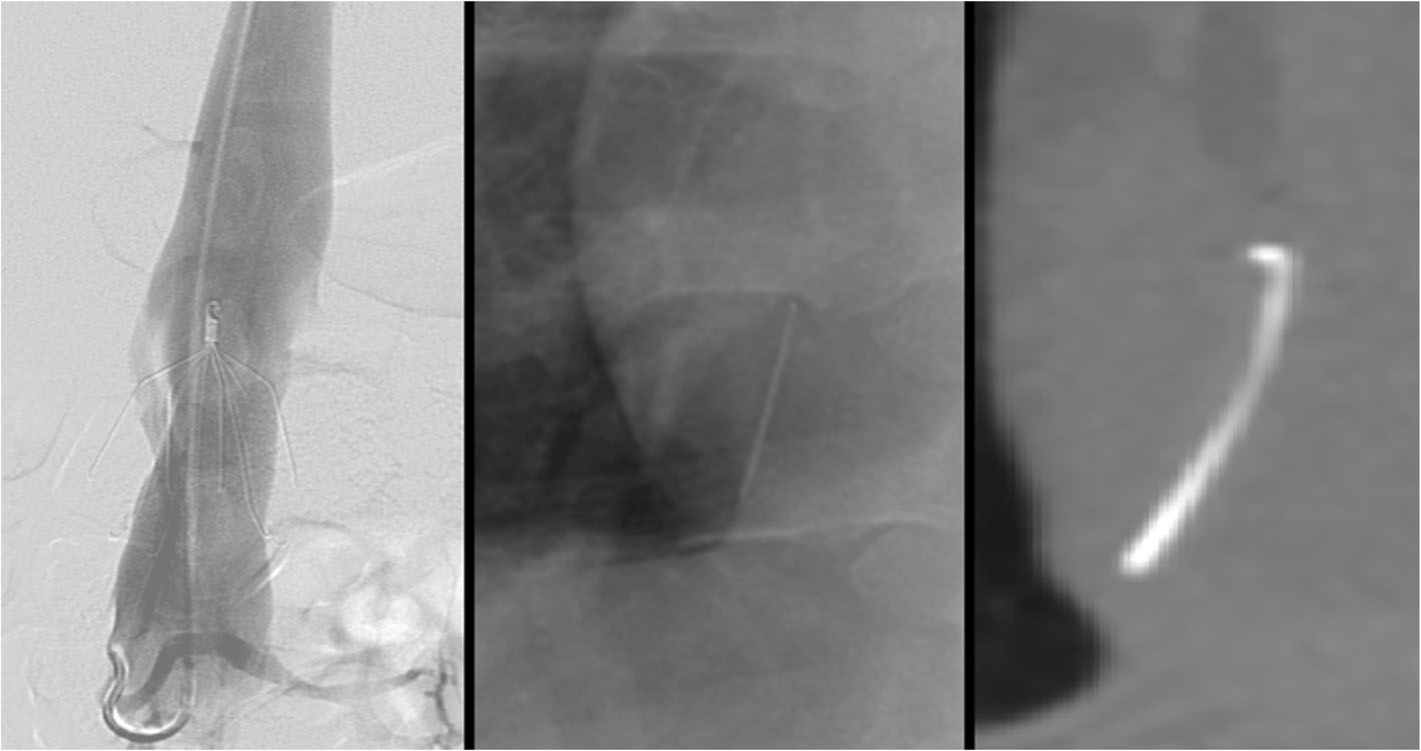
This patient had a pregnancy related DVT and PE and was initiated on a heparin drip. After subsequent C-section, she developed a life threatening abdominal wall hematoma necessitating IVC filter placement. She presented for removal 19 weeks after placement. Initial images showed an intact Bard Eclipse filter (Bard Peripheral Vascular, Inc., Tempe, AZ) with legs penetrating out of the wall of the IVC. After retrieval using the standard loop snare and oversheathing technique, ex-vivo examination showed a missing leg and flouroscopic images showed this to be in the right atrium. As the patient was asymptomatic, retrieval of this fragment was not attempted

Leg penetration > 3 mm is present in 19% of filters but is rarely symptomatic (Grassi et al. 2001; Jia et al. 2015). Some filters have a high rate of leg penetration, for example leg penetration is reported in 39% of Cook Celect filters (Cook, Bloomington, IN) (Zhou et al. 2014). Even if asymptomatic, penetration may increase the rate of retrieval complications. These complications include traumatic injury to the wall of the cava including pseudoaneurysm (Stavropoulos et al. 2015), dissection and intramural hematoma (Al-Hakim et al. 2014). Additionally, caval thrombosis can occur; in a case series of removal of adherent, filters adherent to the wall of the IVC showed a 31% rate of partial caval thrombosis (Fig. 10) (Kuo et al. 2009). Filter leg fractures and embolization can also occur given the increased forces and stresses placed on the filter during removal (Fig. 11). One case report even described embolization of an IVC filter leg down the aorta and into the profunda femoris during retrieval of embedded leg (Knavel et al. 2016). Filter removal with arterial penetration of components using bronchial forceps was shown to be safe in a series of 42 patients (Duncan et al. 2018), however published case reports have also described arteriovenous fistulas after removal (DeSai et al. 2019; Ferral 2019) (see Fig. 2).

##### Prolonged dwell time

Prolonged dwell time, variably defined in the literature from> 90 days or > 180 days, has been associated with higher rates of failure of standard retrieval technique (Geisbusch et al. 2012). Advanced techniques are needed in just over 40% of cases when filters had been in place for over 210 days (Desai et al. 2017). Procedural complications, however, have not been showed to be independently associated with prolonged dwell time; one study that included 52 patients with filters in place for > 6 months showed no increased rates of complications were seen compared to the cohort of patients whose filters were in place for < 6 months (Desai et al. 2015).

## Conclusion

While filter retrieval is generally considered a simple procedure, difficult filter retrievals can be both technically challenging and potentially morbid. Knowing what proceudral techniques and filter/patient related factors are associated with higher rates of complications will help proceduralists anticipate potential complications. Attribution of procedural complications to the use of advanced techniques versus suboptimal filter position is not feasible as advanced techniques are used exclusively for tilted or embedded filters. When removing a “difficult” filter and employing advanced techniques, complications should be anticipated and tamponade balloons as well as appropriate stents/stent-grafts should be immediately available.

## Data Availability

As this is a review paper and case series, no study data is available.

## Abbreviations

DVT: Deep venous thrombosis
HCT: Hematocrit
IVC: Inferior vena cava
PE: Pulmonary embolism
VTE: Venous thromboembolism
